# Supplementary material for: From sequence to enzyme mechanism using multi-label machine learning
Source: BMC Bioinformatics. 2014 May 19;15:150. doi: 10.1186/1471-2105-15-150 (PMC4229970; doi:10.1186/1471-2105-15-150)
Supplement: Additional file 2 — Java code of ml2db. Additional file ml2db_code.tar.gz contains the Java source code to run the multi-label machine learning experiments and save the results to database. The code’s Javadoc is included. [file 1471-2105-15-150-S2.zip › additional file 2/ml2db/ml2db/doc/mulan/classifier/transformation/BinaryRelevance.html]

BinaryRelevance


JavaScript is disabled on your browser.


- Overview
- Package
- Class
- Use
- Tree
- Deprecated
- Index
- Help

- Prev Class
- Next Class

- Frames
- No Frames

- All Classes

- Summary:
- Nested |
- Field |
- Constr |
- Method

- Detail:
- Field |
- Constr |
- Method


mulan.classifier.transformation

## Class BinaryRelevance

- java.lang.Object
- - mulan.classifier.MultiLabelLearnerBase
  - - mulan.classifier.transformation.TransformationBasedMultiLabelLearner
    - - mulan.classifier.transformation.BinaryRelevance

- All Implemented Interfaces:
  :   java.io.Serializable, MultiLabelLearner, weka.core.TechnicalInformationHandler

  ---

    

  ```
  public class BinaryRelevance
  extends TransformationBasedMultiLabelLearner
  ```

  Version:
  :   2012.03.14

  Author:
  :   Robert Friberg, Grigorios Tsoumakas

  See Also:
  :   Serialized Form

- - ### Constructor Summary

    Constructors

    Constructor and Description

    `BinaryRelevance(weka.classifiers.Classifier classifier)`

    Creates a new instance
  - ### Method Summary

    Methods

    | Modifier and Type | Method and Description |
    | --- | --- |
    | `weka.classifiers.Classifier` | `getModel(java.lang.String labelName)` Returns the model which corresponds to the label with labelName |

    - ### Methods inherited from class mulan.classifier.transformation.TransformationBasedMultiLabelLearner

      `getBaseClassifier, getTechnicalInformation, globalInfo`
    - ### Methods inherited from class mulan.classifier.MultiLabelLearnerBase

      `build, getDebug, isUpdatable, makeCopy, makePrediction, setDebug`
    - ### Methods inherited from class java.lang.Object

      `equals, getClass, hashCode, notify, notifyAll, toString, wait, wait, wait`

- - ### Constructor Detail


    - #### BinaryRelevance

      ```
      public BinaryRelevance(weka.classifiers.Classifier classifier)
      ```

      Creates a new instance

      Parameters:
      :   `classifier` - the base-level classification algorithm that will be
          used for training each of the binary models
  - ### Method Detail


    - #### getModel

      ```
      public weka.classifiers.Classifier getModel(java.lang.String labelName)
      ```

      Returns the model which corresponds to the label with labelName

      Parameters:
      :   `labelName` -

      Returns:
      :   the corresponding model or null if the labelIndex is wrong


- Overview
- Package
- Class
- Use
- Tree
- Deprecated
- Index
- Help

- Prev Class
- Next Class

- Frames
- No Frames

- All Classes

- Summary:
- Nested |
- Field |
- Constr |
- Method

- Detail:
- Field |
- Constr |
- Method
